# Functional and Radiological Outcome Analysis of Osteoperiosteal Decortication Flap in Nonunion of Tibia

**DOI:** 10.1155/2021/7980602

**Published:** 2021-07-21

**Authors:** Vineet Kumar, Shah Waliullah, Sachin Avasthi, Swagat Mahapatra, Ajai Singh, Sabir Ali

**Affiliations:** ^1^Department of Orthopaedics, Dr RMLIMS, Lucknow, India; ^2^Department of Orthopaedics, KGMU, Lucknow, India; ^3^Department of Paediatric Orthopaedics, KGMU, Lucknow, India

## Abstract

**Introduction:**

The treatment of long bone shaft nonunions is challenging. The technique of osteoperiosteal decortications flap for approaching the nonunion site coupled with fixation modalities was first described by Judet in 1963. Despite promising clinical and radiological union, this technique is not popular among orthopaedic surgeons. Our study aimed to evaluate the radiological union and functional results of shaft tibia nonunions treated by the osteoperiosteal decortication approach.

**Methods:**

This retrospective study included all the cases with established tibial shaft nonunion following stringent inclusion and exclusion criteria and operated upon by following the principle of osteoperiosteal flap technique from April 2015 to July 2019. Further subgroups were made based on nonunions complexity based on nonunion scoring system (NUSS) score. The outcome measures included radiological union scale in tibial fractures (RUST) and lower extremity functional scale (LEFS). The preoperative scores for union and function were recorded, and the subsequent scores were obtained at three, six, and nine months and one year. Appropriate statistical analysis of the data was done.

**Results:**

Thirty-four cases were shortlisted for analysis, fulfilling our inclusion and exclusion criteria. There were 22 males (64.7%) and 12 females (35.3%) with a mean age of 34.17 ± 10.3 years. Subgroup analysis based on the complexity of nonunion (NUSS score) revealed 14 cases in group A, 10 cases in group B, 10 cases in group C, and 0 cases in group D. The average time from fracture to surgery in these cases was 14.6 months. The average time to achieve union was 9.6 months, with patients in groups A, B, and C, having a mean duration of 9, 10.5, and 12 months, respectively. Statistically, significant improvement was seen in both RUST scores and LEFS score. Complications included infection in seven cases, wound dehiscence in two cases, and four cases of persistent nonunion.

**Conclusion:**

Osteoperiosteal decortication remains a highly effective surgical technique in the management of nonunion of long bones. NUSS scoring is an essential tool for prognosticating nonunion cases. This score is inversely related to the radiological union (RUST score) of the bone and functional recovery (LEFS score) of the patient.

## 1. Background of the Work

The basic fracture healing process is natural, though this is a complex biological process involving bone tissue regeneration. The process of fracture union can well be considered as a variant of tissue regeneration. Under normal circumstances, this bone tissue regenerates, but sometimes it goes into nonunion [1]. The process of fracture union is hampered if there is an insult to the biology of the bone and surrounding tissue. Surgery is a planned iatrogenic insult to the soft tissues. Therefore, it becomes imperative to maintain an adequate balance between soft tissue biology and surgical technique. This balance forms the foundation for the bone regeneration process after the surgery.

Tibia fractures are one of the most typical long bone fractures to go into nonunion. The reasons for the nonunion of tibia have been extensively documented in the literature [2]. The soft tissues surrounding the bones are one of the crucial factors responsible for the fracture healing process. This factor holds even more importance in tibia fractures, as the bone is subcutaneous throughout its anterior and anteromedial aspect. A fresh fracture [3] of the tibial shaft can be managed both surgically and conservatively, but surgical intervention becomes mandatory in cases with established nonunion [4]. The anterolateral approach is the standard surgical approach for addressing the nonunion of the tibia. Several surgical techniques have been described in the literature to address this challenging situation. These techniques are often combined with one or the other bone induction methods to achieve fracture union [5, 6]. When the cause of nonunion is biological, the problem becomes even more challenging to address. The diamond concept introduced by Calori et al. says that there are three biological (growth factors, osteoconductive scaffolds, and osteogenic cells) and a mechanical factor that forms the four pillars required for adequate bone healing during the fracture union process [7, 8]. Therefore, any alteration in any of the factors directly threatens the fracture healing process.

Open surgical procedures disturb the soft tissue envelop surrounding the fracture, more so in the tibial diaphysis, which has already a precarious extraosseous blood supply [9]. The osteoperiosteal decortication flap technique effectively addresses this issue in the nonunion tibia and ensures an adequate biological environment at the nonunion site. Judet first described this technique to manage nonunions of the tibia in 1962, and the results of this technique were first published in 1972 with 92% union results [10]. The bone chips (osteoperiosteal) were denuded from the tibia shaft on either side of the fracture using the standard incision. These bone chips with their blood supply (through the muscles attached) constitute the osteoperiosteal flap. Subsequently, in 2012 Guyver et al., in their publication, demonstrated similar results with 92.3% union rates [11]. Despite promising results, this technique has not gained much popularity. Most of the data available in the literature using this technique have been on the nonunion of long bone fractures. The nonunion of the tibia was exclusively included in this study to have a comparable group on which the outcome analysis would be more justifiable. There are few articles available in the literature, which assess the union rate after Judet's technique. Therefore, we planned this study in cases of the nonunion tibia to validate this technique and assess the functional outcome in cases managed by this technique.

## 2. Materials and Methods

**Figure 1:**
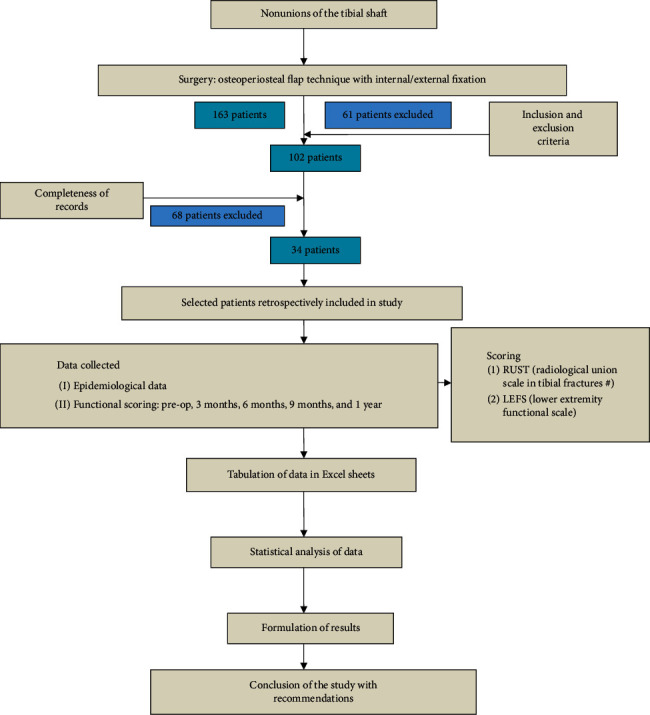
Methodology flowchart.

This retrospective cohort study (Figure 1) was conducted in the Departments of Orthopaedic Surgery at two tertiary care multispecialty teaching hospitals in North India. After approval of the Institutional Ethical Committee (IEC 73/20), a comprehensive data collection was done from the record section of both the institutes from April 2015 to July 2019.

The inclusion criteria comprised all cases aged 12–65 years of either gender with established tibial shaft nonunion [2, 4] operated on using the principle of osteoperiosteal flap technique with fracture stabilization using either an internal or external fixation device.

Cases with neurovascular involvement, musculoskeletal ailment, and any previous surgery in the ipsilateral limb, pregnant females, patients with significant life-threatening comorbidities, patients on immunosuppressive therapies, and nonunion cases requiring a simultaneous plastic procedure were excluded from our study.

We included only the cases with nonunion of the tibia shaft in our series, as the tibia is more prone to go into nonunion amongst all the long bones. This is attributed to its subcutaneous location. The tibia is also more prone to injury in high-velocity injuries [12–15]. The incidence of nonunion in the tibia further increases in cases of open fracture.

The study sample was further categorized into four groups based on the complexity of nonunion, as per the Calori nonunion scoring system (NUSS) criteria devised by Calori et al. [16, 17]. NUSS is a complex scoring system with eighteen variables summing up to a maximum total score of 50. This score is then doubled to 100 and divided equally in four groups, which signify the severity of nonunion. The primary outcome measures were to analyze the radiological union and the functional status of the limb. The radiological union scale in tibial fractures (RUST) score was used for the estimation of radiological union, and the lower extremity functional scale (LEFS) was used for the assessment of functional status [18–23]. The RUST scoring system (range = 4–12) utilises X-rays in both anteroposterior and lateral views to assess union by documenting the bridging callus and visible fracture line in all four cortices. The LEFS scoring system (range = 0–80) is a questionnaire (20 questions) for assessing the functional state of the lower extremity, with each question carrying a score from 0–4.

The radiological union and function scores were recorded preoperatively and, after that, subsequently at three months, six months, nine months, and one year. The data were recorded on the excel sheet and analyzed. The results obtained were compared with the data available in the literature. The outcome analysis was also done between the groups categorized as per the Calori system to assess this procedure's efficacy. Statistical analysis of the data was done using GraphPad Prism version 7 (GraphPad Software, San Diego, CA, USA).

## 3. Results

We could retrieve 34 cases for analysis from the database, fulfilling our inclusion and exclusion criteria. Of these, there were 22 males (64.7%) and 12 females (35.3%), with a mean age of 34.17 ± 10.3 years, and road traffic accident (RTA) was the most common cause of primary injury (Table 1). The patients were further analyzed by categorizing them into four groups to have similarity in the fracture's complexity pattern as per the Calori scoring system. The categorization was done to minimize the bias during the analysis stage. We had 14 cases in group A, 10 cases in group B, and 10 cases in group C, while no patients were available in group D. The average time from fracture to surgery in these cases was 14.6 months (*R* = 9–24 months). The follow-up records of all the cases were obtained for one year. The average time to achieve union was 9.6 months, with patients in groups A, B, and C, having a mean duration of 9, 10.5, and 12 months, respectively (*A* = 7.92 ± 1.49, *B* = 10.5 ± 1.58, *C* = 11.1 ± 1.44). Data were analyzed within the respective groups using a “paired *T*-test” with 95% CI of difference of means and were recorded for their RUST score (Table 2) and LEFS score (Table 3) at the predetermined intervals.

As evaluated by the RUST score, the radiological union in groups B and C demonstrated a statistically significant improvement from six months postoperatively. In contrast, group A showed a statistically significant improvement from three months postoperatively (Table 2). The functional status in groups A and B, as evaluated by the LEFS score, demonstrated a statistically significant improvement from three months postoperatively. In contrast, group C showed a statistically significant improvement from six months postoperatively (Table 3). Intergroup analysis of the mean RUST scores and LEFS scores using ANOVA revealed a significant improvement at each visit from three months onwards (Table 4).

The complications encountered in our study included infection, persistent nonunion, and wound dehiscence. Postoperative infection was seen in seven cases. One of them required debridement of the wound and a plastic procedure, whereas the remaining cases were successfully managed with an extended regime of antibiotics. Out of the seven infected cases, five cases were from group C. The statistical analysis of the data (chi-square test) revealed a significantly increased postoperative infection rate in group C (*P*=0.0232). In addition, we had four cases with persistent nonunion (cases where we could not achieve union at 12 months), of which one case was from group B and three cases from group C. However, on intergroup analysis, the difference was not found to be statistically significant (*P*=0.0781). Finally, we had two cases of wound dehiscence, one each from groups B and C (Table 5). Both of them required debridement of the wound followed by a plastic surgery procedure.

Resurgery was required in 29% (10/34) of the cases, of which eight cases were from group C and one each from groups A and B. Resurgery included plastic procedure or bone grafting. 80% (8/10) of the cases in group C required resurgery. Intergroup chi-square analysis revealed a statistically significant increase in the requirement for resurgery in group C (*P*=0.0125) (Table 6).

Correlation analysis between NUSS versus RUST score and NUSS versus LEFS score at subsequent follow-up using Spearman *r* correlation showed statistically significant negative correlation, except the preoperative RUST (*P*=0.1286) and LEFS (*P*=0.1540) score (Table 7).

## 4. Discussion

Nonunions, though common, are tricky to address. Comprehensive data are available in the literature for the management of the same. The use of adjuvants and the type of implant used, rather than soft tissue biology, are usually prioritized in the management of these nonunions. The focus of the current study is the soft tissue dissection element. For orthopaedic surgeons, fresh diaphyseal tibia fractures are simple to treat, but nonunions of these fractures pose a significant challenge. Fresh diaphyseal tibial fractures have shown a good outcome in conservatively managed cases with a nonunion rate close to 1.1%. In contrast, the literature reports a nearly 5% nonunion rate in operatively managed cases [8, 24]. This calls for some insight into the blood supply of diaphysis of the tibia. The major vascularity in the tibial diaphysis is by the tibial nutrient artery (TNA), which is responsible for supplying the inner two-third of the diaphyseal cortex [9]. The extraosseous blood supply of the tibial diaphysis is poor compared with the proximal and distal metaphysic [25]. This is probably the reason for increased nonunion rates in metaphyseal areas if managed by open reduction and plating. Therefore, results are better in these cases when managed by minimally invasive percutaneous plate osteosynthesis (MIPPO), as this technique utilises the submuscular plane instead of subperiosteal dissection. In cases of established nonunion, open reduction is the only alternative. Judet's osteoperiosteal flap technique provides a possible solution for these fractures. The osteoperiosteal flap technique, described initially by Judet and Patel, has been used as a soft tissue handling technique to manage aseptic nonunions. This technique avoids the subperiosteal plane and elevates the soft tissue with the cortical bone underlying the periosteum. These flaps are with the muscle attachments, which have a blood supply that aids in new bone production and, eventually, union at the fracture site. Judet and Patel reported a 92% success rate in their series of 1068 cases [10]. In his series, there were 290 cases of aseptic nonunion with 94.8% union rates, 126 cases of septic nonunion with 85% union rates and 108 malunion cases with 98% union rates. In his latest series of 297 cases, he reported a union rate of 99% in eight months [26].

Our surgical management protocol involved the denudation of the superficial cortical bone with a sharp osteotome along with the soft tissue flap for exposure of the nonunion site. This technique helps in retaining the vascularity of the flap and thus prevents skin necrosis. The cortical bone chips also provide a local graft at the nonunion site for enhancing union, although this bone is not considered adequate in cases of nonunion with bone defects [27]. This dissection technique also ensures that the surgical wound heals adequately and quickly. The sutures also have a firm hold due to the cortical bone chips adhered to the soft tissue envelope, thus preventing wound dehiscence despite friable and inadequate tissue at the local site. This is of importance in cases of resurgery or in cases having a previous open wound scar or a plastic procedure. However, this procedure may not be suitable to address periarticular and intra-articular fracture nonunions due to the absence of periosteal cover.

A few studies have used Judet's technique for addressing the nonunion. Ramoutar et al. [27] showed a 95% union rate in his case series using the Judet technique to treat nonunion of both upper and lower limb bones. They also advocated that proper execution of the Judet technique was associated with a decrease in the requirement of autologous bone graft for treatment of the nonunion. We considered osteoperiosteal flap as an adjunctive procedure for an extra bone graft at the nonunion site. In this series, we used autologous bone graft in almost all of our cases. We had four cases of persistent nonunion, that is, 88.2% (30/34) union rate in our series. The union rate was 100% in group A, 90% in group B, and 70% in group C. Thus, the union rates in our cases vary from 70%–100%, depending on the initial NUSS score with which the patient has presented. Thus, NUSS scoring system appears to have prognostic importance in cases of fracture nonunions. The disparity in results among the three groups underlines the need of categorizing nonunions based on their severity or complexity before prognosticating the case and estimating the likelihood of union. Guyver et al. [11] observed a 92% union rate in their study, while Raju et al. [28] showed a 100% union rate in their case series of 20 patients with tibial fracture nonunion. In both these studies, the classification of nonunion according to their severity or complexity has not been taken into consideration. Guyver observed three patients with superficial infections and two patients with deep infections [11]. In our study, we had an infection rate of 20.5% (7/34), out of which the infection rate in group A was 7% (1/14), 10% (1/10) in group B, and 50% (5/10) in group C. The infection rate was significantly higher in group C compared with groups A and B. This increased infection rate could be attributed to the factors we considered in NUSS scoring. We were able to manage infection in all the cases with an extended antibiotic regime, except in one case in group C, which required wound debridement and subsequent plastic procedure.

In the current study, the radiological union was evaluated by RUST score. The RUST scoring system, with its advent way back in 2010, has shown a formidable performance with excellent intra- and interrater reliability for grading union and predicting union in tibial shaft fractures [18–21]. For clinical evaluation, we chose the LEFS score. LEFS has been shown to have good reliability and predictive correlation in assessing lower limb. Moreover, it is a reliable and valid tool for monitoring recovery in cases with tibia shaft fractures [22, 23].

**Figure 2:**
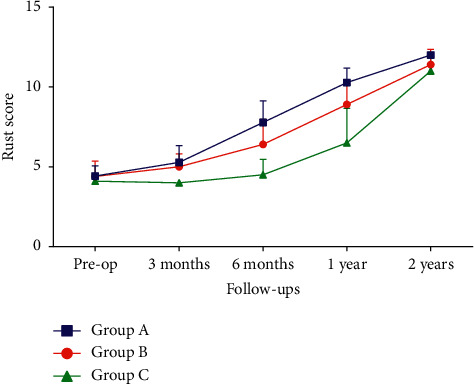
Graphical representation showing RUST score at subsequent follow-up of patients of different groups.

**Figure 3:**
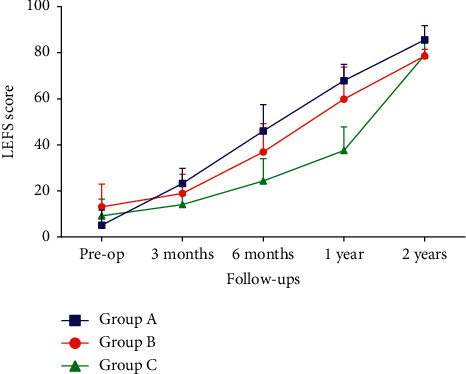
Graphical representation showing LEFS score at subsequent follow-up of patients of different groups.

We observed that the average time taken for the radiological union was similar to the graph pattern of clinical improvement as evaluated by LEFS (Figures 2 and 3). Thus, we performed a correlation analysis. This analysis revealed a strong correlation between NUSS and RUST/LEFS score from the third month postoperatively. The lesser the NUSS score, the better the union rate and the functional outcome as interpreted by the RUST score and LEFS score. This association is best observed between three and nine postoperative months. Prognostication in terms of the time to union in relation to the NUSS score is thus well explained by the correlation analysis.

Limitations of our study included the small sample size and absence of a control group. We have used autologous bone graft in almost all cases. We, therefore, could not comment on the usefulness of local graft alone, which is created by decortication for promoting union at the nonunion site. The types of nonunion (atrophic, oligotrophic, and hypertrophic) have not been categorized and analyzed separately. They have been taken as a part of the scoring system (NUSS). Similarly, aseptic and septic cases have also not been analyzed separately. However, in our series, 71.5% (5/7) cases with clinical signs of infection landed up in group C after the NUSS score. One more important limitation of this study is that we have not considered and have not standardized the nonunion fixation or stabilization method. Standardization of the fracture fixation method could have further added new information in managing nonunion cases by this technique.

## 5. Conclusion

Judet's technique of osteoperiosteal decortication combined with autologous corticocancellous bone grafting and internal or external fracture stabilization device is a highly effective and reproducible surgical technique in the management of diaphyseal fracture nonunion. NUSS scoring is an essential tool for prognosticating nonunion cases. This score is inversely related to the radiological union (RUST score) of the bone and functional recovery (LEFS score) of the patient.

## Figures and Tables

**Table 1:** Demographic details of the study population.

Parameters		Group A NUSS (1–25) [*n* = 14]	Group B NUSS (26–50) [*n* = 10]	Group C NUSS (51–75) [*n* = 10]	Total [*n* = 34]
Age (in years)		35 ± 10.82	31.6 ± 9.14	35 ± 10.94	34.17 ± 10.3
		35	31.5	36	

Sex	Male	8	6	8	22 (64.7%)
	Female	6	4	2	12 (35.3%)

Mode of injury	RTA	10	9	8	27 (79.4%)
	Assault	3	0	2	5 (14.7%)
	Fall from height	1	1	0	2 (5.9%)

**Table 2:** Comparison of RUST score at serial follow-up.

RUST score	Group A NUSS (1–25) [*n* = 14]		Group B NUSS (25–50) [*n* = 10]		Group C NUSS (51–75) [*n* = 10]	
	95.00% CI of diff.	*P* value	95.00% CI of diff.	*P* value	95.00% CI of diff.	*P* value
Pre-op vs. 3 months	−1.833 to 0.1132	0.1080	−2.129 to 0.9295	0.7980	−1.48 to 1.68	0.9998
3 months vs. 6 months	−3.473 to −1.527	<0.0001^*∗*^	−2.929 to 0.1295	0.0872	−2.08 to 1.08	0.8958
6 months vs. 9 months	−3.473 to −1.527	<0.0001^*∗*^	−4.029 to −0.9705	0.0003^*∗*^	−3.58 to −0.4196	0.0068^*∗*^
9 months vs. 12 months	−2.693 to −0.7468	<0.0001^*∗*^	−3.379 to −0.3205	0.0107	−6.08 to −2.92	<0.0001^*∗*^

^*∗*^Significant; Student's *t*-test.

**Table 3:** Comparison of LEFS at serial follow-up.

LEFS score	Group A NUSS (1–25) [*n* = 14]		Group B NUSS (25–50) [*n* = 10]		Group C NUSS (51–75) [*n* = 10]	
	95.00% CI of diff.	*P* value	95.00% CI of diff.	*P* value	95.00% CI of diff.	*P* value
Pre-op vs. 3 months	−26.4 to −9.741	<0.0001^*∗*^	−18.74 to 7.139	0.7083	−15.36 to 5.565	0.6740
3 months vs. 6 months	−31.12 to −14.46	<0.0001^*∗*^	−30.94 to −5.061	0.0024^*∗*^	−20.66 to 0.265	0.0593
6 months vs. 9 months	−30.18 to −13.52	<0.0001^*∗*^	−35.84 to −9.961	<0.0001^*∗*^	−23.66 to −2.735	0.0070^*∗*^
9 months vs. 12 months	−25.98 to −9.321	<0.0001^*∗*^	−31.64 to −5.761	0.0015^*∗*^	−51.96 to −31.04	<0.0001^*∗*^

^*∗*^Significant; Student's *t*-test.

**Table 4:** Intergroup analysis of mean RUST and LEFS score.

		Group A NUSS (1–25) [*n* = 14]	Group B NUSS (25–50) [*n* = 10]	Group C NUSS (51–75) [*n* = 10]	*P* value
RUST score	Pre-op	4.42 ± 0.64	4.4 ± 0.96	4.1 ± 0.31	*F* = 0.736
		4	4	4	*P* = 0.487
	3 months	5.28 ± 1.06	5 ± 0.81	4 ± 0	*F* = 7.56
		5	5	4	*P* **=** **0.0021**^*∗*^
	6 months	7.78 ± 1.36	6.4 ± 1.57	4.5 ± 0.97	*F* = 17.78
		8	6	4	*P* **<** **0.0001**^*∗*^
	9 months	10.28 ± 0.91	8.9 ± 1.28	6.5 ± 2.17	*F* = 19.9
		10	8.5	6.5	*P* **<** **0.0001**^*∗*^
	12 months	12 ± 0	10.75 ± 1.25	11 ± 1.41	*F* = 5.207
		12	11	11.5	*P* **=** **0.0112**^*∗*^

LEFS score	Pre-op	5.14 ± 6.61	13.1 ± 9.84	9.2 ± 7.22	*F* = 3.033
		2	13.115.5	9	*P* = 0.0626
	3 months	23.21 ± 6.61	18.9 ± 8.25	14.1 ± 5.30	*F* = 5.255
		20	20.5	15	*P* **=** **0.0108**^*∗*^
	6 months	46 ± 11.50	36.9 ± 12.35	24.3 ± 9.67	*F* = 28.08
		43.5	37.5		*P* **<** **0.0001**^*∗*^
	9 months	67.85 ± 7.11	59.8 ± 13.88	37.5 ± 10.27	*F* = 25.63
		69.5	62.5	35.5	*P* **<** **0.0001**^*∗*^
	12 months	85.5 ± 6.18	78.5 ± 2.88	79 ± 7.74	*F* = 5.256
		87.5	78.5	81	*P* **=** **0.0108**^*∗*^

^*∗*^Significant; ANOVA test.

**Table 5:** Complications.

Complications		Group A NUSS (1–25) [*n* = 14]	Group B NUSS (25–50) [*n* = 10]	Group C NUSS (51–75) [*n* = 10]	*P* value
Infection	Yes	1	1	5	χ = 7.525
	No	13	9	5	*P* **=** **0.0232**^*∗*^

Wound dehiscence	Yes	0	1	1	χ = 1.488
	No	14	9	9	*P* = 0.4753

Nonunion	Yes	0	1	3	χ = 5.1
	No	14	9	7	*P* = 0.0781

**Table 6:** Patients requiring resurgery.

Resurgery required	Group A NUSS (1–25) [*n* = 14]	Group B NUSS (25–50) [*n* = 10]	Group C NUSS (51–75) [*n* = 10]	*P* value
Yes	1	1	8	χ = 8.762
No	13	9	2	*P* **=** **0.0125**^*∗*^

^*∗*^Significant. χ, chi-square test.

**Table 7:** Correlation analysis of NUSS versus RUST and LEFS at various follow-ups.

NUSS vs.	RUST score	RUST (1^st^ FU)	RUST (2^nd^ FU)	RUST (3^rd^ FU)	RUST (4^th^ FU)
Spearman *r*	−0.2659	−0.5558	−0.7583	−0.7951	−0.5513
95% confidence interval	−0.561 to 0.0897	−0.757 to −0.2584	−0.8751 to −0.5579	−0.8952 to −0.6186	−0.8422 to −0.01182
*P* (two-tailed)	**0.1286**	**0.0006** ^*∗*^	**<0.0001** ^*∗*^	**<0.0001** ^*∗*^	**0.0444** ^*∗*^

NUSS vs.	LEFS	LEFS (1^st^ FU)	LEFS (2^nd^ FU)	LEFS (3^rd^ FU)	LEFS (4^th^ FU)
Spearman *r*	0.2862	−0.5574	−0.7145	−0.7699	−0.4016
95% confidence interval	−0.067 to 0.576	−0.758 to −0.2605	−0.8507 to −0.4884	−0.8815 to −0.5767	−0.7755 to 0.1809
*P* (two-tailed)	**0.1008**	**0.0006** ^*∗*^	**<0.0001** ^*∗*^	**<0.0001** ^*∗*^	**0.1540**

^*∗*^Significant; Spearman *r* correlation.

## Data Availability

The retrospective clinical data used to support the findings of this study are included within the article.
